# An SRR1 domain-containing protein is required for efficient Orsay virus replication in *Caenorhabditis elegans*

**DOI:** 10.1128/jvi.00521-25

**Published:** 2025-09-03

**Authors:** Chika Fujii, David Wang

**Affiliations:** 1 Department of Molecular Microbiology, School of Medicine, Washington University in St. Louis 7548, St. Louis, Missouri, USA; 2 Department of Pathology and Immunology, School of Medicine, Washington University in St. Louis 7548, St. Louis, Missouri, USA; University of Kentucky College of Medicine, Lexington, Kentucky, USA

**Keywords:** *Caenorhabditis elegans*, virus replication, host factor

## Abstract

Viruses depend on their hosts for completing their life cycle, and a better understanding of virus replication can inform therapeutic strategies. Using the Orsay virus-*Caenorhabditis elegans* experimental platform, we identified by a forward genetic screen the host gene *Y55F3BL.4* (renamed *viro-9*) as a novel host factor critical for Orsay virus replication. Three distinct mutations of *viro-9* each resulted in a >1,000-fold reduction in Orsay viral load, demonstrating a pro-viral function of *viro-9. viro-9* had no previously described function in *C. elegans*, but in the absence of viral infection, deletion of the *viro-9* locus by CRISPR/Cas9 led to a reduction in brood size and a shortened lifespan. VIRO-9 contains a *s*ensitivity to *r*ed light *r*educed (SRR1) protein domain. While SRR1 domains are present in diverse organisms, including plants, yeast, and mammals, little is known about their function. The *Caenorhabditis briggsae* ortholog of *viro-9*, *CBG23913*, can functionally complement the *C. elegans viro-9* defect, demonstrating that the pro-viral function of the SRR1 domain is conserved over at least 80 million years of evolution. Furthermore, we identified three conserved amino acid residues within the SRR1 domain that are required for Orsay virus infection. This study provides the first insights into amino acids necessary for functionality of the SRR1 domain and demonstrates the essential role of *viro-9* in virus infection.

**IMPORTANCE:**

Host factors required for viral replication could serve as therapeutic targets for various viral species. The *Caenorhabditis elegans-*Orsay virus experimental system offers a platform for identifying genes important for virus infection in nematodes that may also be important for human-infecting viruses. We determined that *viro-9*, a previously uncharacterized gene in *C. elegans* containing the SRR1 domain, is required for Orsay virus replication. The related gene in *Caenorhabditis briggsae*, a relative of *C. elegans* that diverged about 80 million years ago, can substitute for *viro-9*, demonstrating that this protein’s ability to promote virus replication is functionally conserved. Because SRR1 domain-containing proteins exist in nematodes, fungi, *Drosophila*, plants, and mammals, including humans, these proteins could be important for facilitating virus infection in other organisms as well.

## INTRODUCTION

Viruses depend on their host to complete their life cycle. A better understanding of host factors involved in the replication step will facilitate the development of antiviral therapeutics and medical intervention strategies. The replication phase of virus infection is an important step to target. Many clinically approved antiviral agents target virus replication by acting as nucleotide or nucleoside analogs, thereby blocking the viral polymerase function (1–4). Having a detailed understanding of fundamental aspects of viral replication could thus offer further insights into ways to limit virus infection. For example, pro-viral host factors necessary for virus replication could be future targets for antiviral development.

Orsay virus is a non-enveloped, bi-segmented, positive-sense, single-stranded RNA virus, which was originally isolated from *Caenorhabditis elegans* in the wild (5). The *C. elegans*-Orsay virus experimental system provides a unique opportunity to study host-virus interactions in a multicellular host. The ~1-mm size of the *C. elegans* nematode permits culturing at a large scale, and it has been successfully leveraged for high-throughput forward genetic screens (6). In prior studies, we identified multiple evolutionarily conserved genes that are critical for virus infection, including *sid-3* (7)*, viro-2* (7)*, nck-1* (7)*, drl-1* (8)*, hipr-1* (9)*,* and *alg-1* (10). These host factors were identified in a genetic screen based on a *C. elegans* reporter strain, *jyIs8; rde-1* (11), which harbors an integrated *gfp* transgene regulated by the promoter of *pals-5*, a virus response gene that is highly upregulated upon Orsay virus infection (12). The *rde-1* mutation renders the *C. elegans* hypersusceptible to Orsay virus infection, thereby ensuring a GFP-on rate close to 100% upon virus infection. The chemical mutagenesis screen selected for mutants failing to express GFP, which are unable to support virus infection. Importantly, the mammalian orthologs of *sid-3*, *viro-2*, *nck-1*, and *hipr-1* have been shown to play a critical role during picornavirus infection in mammalian cells (9, 13). Additionally, the human ortholog of *alg-1*, Argonaute 2, has been shown to be important in stabilizing the viral genome to facilitate hepatitis C virus replication (14). These studies highlight the evolutionary conservation of host gene function of factors isolated from a *C. elegans* screen.

The *s*ensitivity to *r*ed light *r*educed (SRR1) Pfam-annotated domain (15) is present in proteins encoded by genes of different organisms in the Animalia, Fungi, and Plantae kingdoms. In yeast, the *ber1* gene containing the SRR1 domain has been described to play a role in microtubule organization and maintaining chromosome stability (16, 17). *srr1*, the *Arabidopsis thaliana* gene containing the SRR1 domain, has been shown to be important for regulating circadian rhythm genes (18). Additionally, *Arabidopsis thaliana srr1* mutants exhibit a reduction in red light sensitivity due to impairments in phytochrome B-mediated photoreceptor signaling (18). As a result, mutant plants fail to accumulate normal chlorophyll levels and flower earlier than wild-type plants (18). In *C. elegans,* there is a single gene, *Y55F3BL.4* (renamed *viro-9* below), that contains an SRR1 domain. Its biological functions in *C. elegans* are unknown, and no explicit studies of this gene have been reported to our knowledge. *Caenorhabditis briggsae*, a *Caenorhabditis* nematode species that diverged from *C. elegans* approximately 80–110 million years ago, encodes one ortholog of *C. elegans viro-9*, *CBG23913* (19). VIRO-9 shares 56% amino acid identity with CBG23913. Humans and mice encode one gene containing the SRR1 domain, the *SRR*1 *d*omain containing (*SRRD*). While the biochemical function of SRRD remains to be characterized, murine SRRD plays a role in blood cell proliferation (20), while human SRRD is implicated in the formation of the aggresome and organization of the intermediate filament (21). In a genome-wide CRISPR/Cas9 screen aimed to identify modifiers of the SQSTM1, an adapter protein implicated in autophagy and that regulates different cellular signaling pathways, human *SRRD* was isolated together with known post-translational modification pathways such as the ubiquitin-proteasome pathway and ufmylation pathway (22). In the context of virus infection, mammalian cell culture CRISPR/Cas9 screens have uncovered *SRRD* as a pro-viral host factor for hepatitis C virus (23) and SARS-CoV-2 (24, 25) infection, although the biochemical mechanism of SRRD in virus infection is currently unknown, and detailed characterization of SRRD is lacking.

Here, we describe a novel genetic screen in *C. elegans* that targets host factors important for the replication stage of the Orsay virus life cycle. The transgenic *C. elegans* line, WUM29, harbors chromosomally integrated plasmid-based sequences of the Orsay virus RNA1 and RNA2 genome segments under control of the heat shock promoter, *hsp-16*, in the *jyIs8; rde-1* reporter background (7). In this strain, the Orsay virus life cycle can be initiated by subjecting the animals to heat shock, thereby bypassing the viral entry stage and focusing the output of the screen on post-entry host factors. Because Orsay virus replication is sufficient to activate the *pals-5*::GFP reporter, this screen can identify mutations in host factors that impact viral replication. Using this approach, we identified *viro-9* as a novel *C. elegans* host factor important for Orsay virus replication. We found that the *C. briggsae* ortholog *CBG23913* can rescue Orsay virus infection, suggesting an evolutionarily conserved role of *viro-9* in the context of virus infection. Furthermore, we identified critical conserved amino acids within the SRR1 domain that are necessary for its pro-viral role.

## RESULTS

### An unbiased EMS mutagenesis screen targeting replication host factors

**Fig 1:**
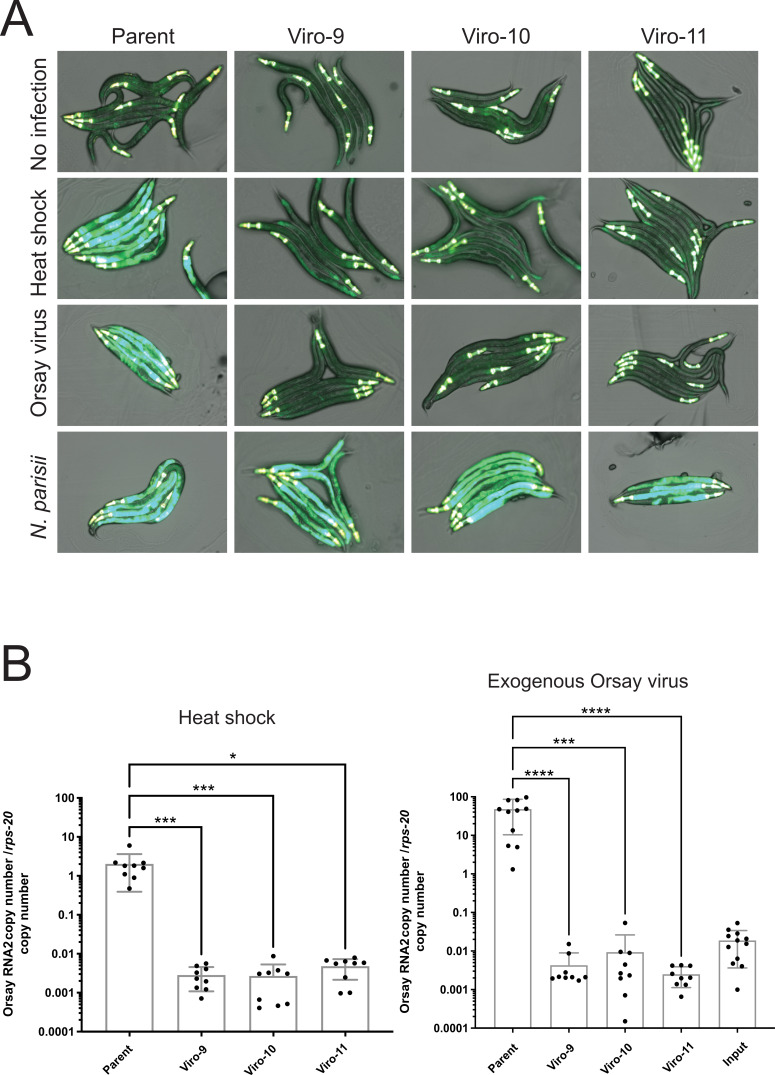
Initial characterization of virus reporter expression and virus load among mutant animals isolated from the chemical mutagenesis screen. (**A**) GFP expression pattern upon heat shock, infection with *N. parisii*, or exogenous Orsay virus among candidate virus reporter-off mutants. (**B**) Quantification of Orsay virus RNA2 upon exogenous Orsay virus infection or initiating the virus life cycle by heat shock. Each bar represents the mean of three independent experiments, and error bars represent the SD. Three independent experiments were performed. Kruskal-Wallis test was performed, followed by Dunn’s multiple comparison test. Adjusted *P*-values are reported as follows: *, *P* < 0.05; ***, *P* < 0.001; ****, *P* < 0.0001. Non-significant *P*-values are not shown.

To identify *C. elegans* host factors involved in Orsay virus replication, WUM29 *virIs1; jyIs8; rde-1(ne219*) animals were screened at a scale of 6,000 haploid genomes. WUM29 animals were subjected to ethyl methanesulfonate (EMS) treatment, which predominantly induces G to A nucleotide substitution mutations into the genome of the progeny generation (26). Subsequently, Orsay virus infection was initiated by activating the integrated Orsay virus transgene, and we screened for mutants that failed to express GFP fluorescence. From this screen, three independent virus reporter off (Viro) strains, Viro-9, Viro-10, and Viro-11, were isolated, all of which had a defect in the expression of GFP (Fig. 1A). In contrast, mutants expressed GFP upon infection with *Nematocida parisii*, a fungal pathogen that was previously described to infect *C. elegans* that also induces *pals-5* expression (27). This indicated that the lack of GFP expression in Viro mutants was Orsay virus-specific (Fig. 1A). The three Viro mutants exhibited between 420- and 760-fold reductions in Orsay virus RNA levels as quantified by qRT-PCR, upon initiating the Orsay virus life cycle intracellularly (Fig. 1B). The low virus RNA copy number in Viro mutants compared to the parental strain after heat shock reflects a deficiency in virus replication. Furthermore, *de novo* exogenous Orsay infection of these strains led to a 15,000- to 39,000-fold reduction in Orsay virus load, indicating that the isolated Viro mutants were also unable to support authentic Orsay virus infection (Fig. 1B).

### *viro-9* is the gene responsible for the lack of GFP and low virus load in Viro mutants

**Fig 2:**
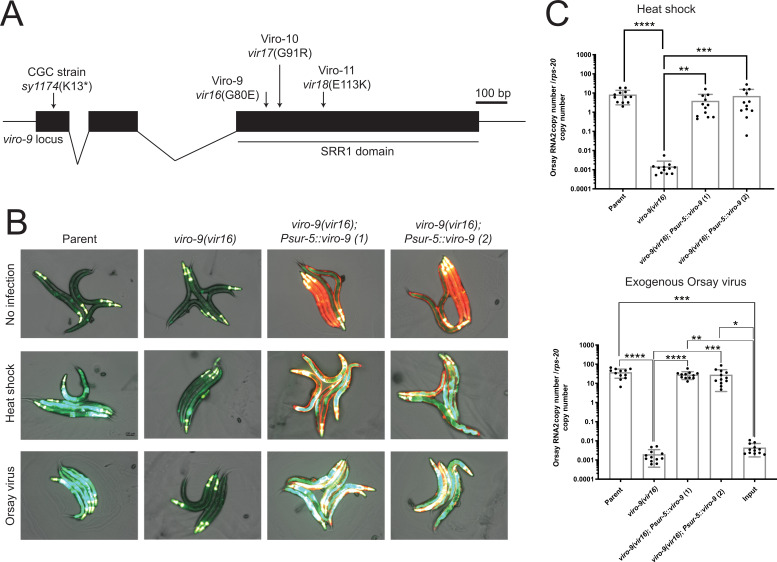
*Viro-9* mutant alleles, GFP expression pattern, and virus load. (**A**) Schematic of the *viro-9* locus. Mutant allele positions and identities are indicated with arrows. (**B**) GFP expression pattern among rescue lines in which *viro-9* is expressed from an overexpression transgene driven by the *Psur-5* promoter. (**C**) qRT-PCR quantification of Orsay virus RNA2 upon exogenous Orsay virus infection or the initiation of the Orsay virus life cycle by heat shock. Each bar represents the mean of the sample, and error bars represent the SD. Three independent experiments were performed. Kruskal-Wallis test was performed, followed by Dunn’s multiple comparison test. Adjusted *P*-values are reported as follows: *, *P* < 0.05; **, *P* < 0.01; ***, *P* < 0.001; ****, *P* < 0.0001. Non-significant *P*-values are not shown.

To identify the gene responsible for the virus replication defect, we took two independent approaches to analyze the genetic lesion in the Viro mutants. First, we sequenced the genomes of Viro-9, Viro-10, and Viro-11. Only one gene, *Y55F3BL.4*, had mutations in all three strains; G-to-A substitutions leading to G80E, G91R, and E113K amino acid substitutions in Viro-9, Viro-10, and Viro-11 strains, respectively, were detected (Fig. 2A). For the second approach, we performed F2 bulk segregation analysis using the Viro-9 strain as described previously (7). We crossed Viro-9 to the unmutagenized parent and expanded single F2 progeny. After a challenge with the Orsay virus, we pooled the DNA of F2 populations that failed to activate GFP and sequenced the pooled DNA. Linkage analysis identified a genomic locus on chromosome IV, linked to the GFP-off phenotype. This region encompassed the *Y55F3BL.4* gene and contained the G80E mutation (Fig. 2A). Based on these results, the *Y55F3BL.4* gene was renamed to *viro-9*. Since the three strains harbor distinct alleles of the same gene, the mutant animals are referred to hereafter as: Viro-9 = *viro-9(vir16*), Viro-10 = *viro-9(vir17*), and Viro-11 = *viro-9(vir18*).

To further validate that the mutation in *viro-9* was the cause of the Orsay virus defect, an overexpression construct containing wild-type *viro-9* was introduced into *viro-9(vir16*) to generate two independent transgenic lines. Upon initiating the virus life cycle intracellularly by heat shock or infecting with exogenous Orsay virus, GFP expression was restored (Fig. 2B), and Orsay virus RNA2 levels were comparable to those of the unmutagenized parental strain (Fig. 2C). These data demonstrated that *viro-9* was the causal gene that was important for virus replication. To provide additional validation, we obtained a mutant strain *Y55F3BL.4*(*sy1174*) from the Caenorhabditis Genetics Center (CGC), which contained a premature stop codon at the 13th amino acid of *viro-9* (Fig. 2A). Upon exogenous Orsay virus infection, we found an ~8,200-fold reduction in Orsay virus load compared to the reference *N2* strain (Fig. S1). This result further confirmed that *viro-9* is important for Orsay virus infection.

### Characterization of basic life history traits of *viro-9* mutant animals

**Fig 3:**
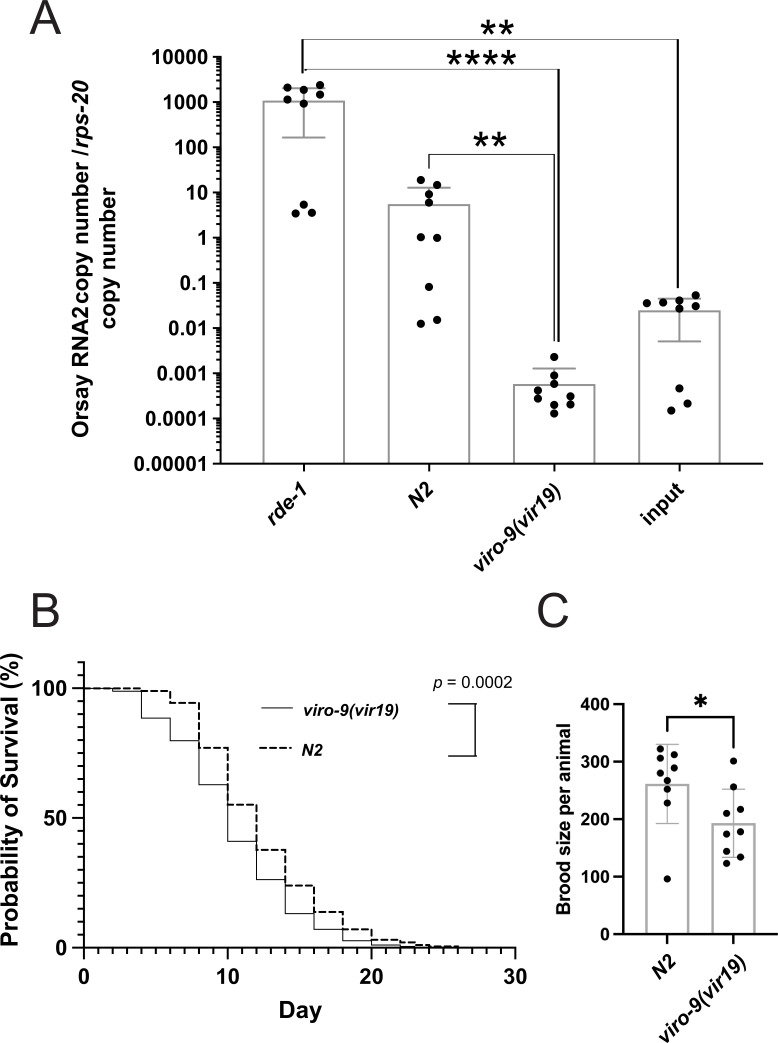
Characterization of life history traits of *viro-9(vir19*) mutant animals generated by CRISPR/Cas9. (**A**) qRT-PCR quantification of Orsay virus RNA2 copy number upon infection with exogenous Orsay virus. Each bar represents the mean of the sample, and error bars represent the SD. Three independent experiments were performed. Kruskal-Wallis test was performed, followed by Dunn’s multiple comparison test. Adjusted *P*-values are reported as follows: **, *P* < 0.01; ****, *P* < 0.0001. Non-significant *P*-values are not shown. (**B**) Survival probability curve comparing *viro-9(vir19*) and the parental *N2* strain. Median survival times were 10 days and 12 days for *viro-9(vir19*) and *N2*, respectively. The log-rank test was performed to determine statistical significance, where *P* = 0.0002 (***). (**C**) Brood size of *viro-9(vir19*) and parental *N2* strain. The Mann-Whitney test was used for statistical analysis of the brood size, where *P* = 0.03 (**).

Since *viro-9* has not been studied in *C. elegans,* we assessed some of the basic life history traits of *viro-9* mutant animals in the absence of virus infection. We generated a complete null allele in the reference *N2* background of *viro-9* by removing the genomic locus, including all three exons, by CRISPR/Cas9 editing. The resulting mutant, *viro-9(vir19*), was backcrossed four times to the parental *N2* reference strain to account for any off-target effects of the CRISPR/Cas9 editing. We first addressed how the complete null allele in *viro-9(vir19*) affected Orsay virus replication. Upon infection with exogenous Orsay virus, the virus load was 9,600-fold lower in *viro-9(vir19*) animals compared to the reference *N2* (Fig. 3A), consistent with the low viral load in *viro-9* mutants isolated from the chemical mutagenesis screen (Fig. 1B). To address whether the loss of *viro-9* had any impact on *C. elegans* life history traits, we interrogated the lifespan and brood size. We found that *viro-9(vir19*) animals exhibited a shortened median survival time of 10 days, in contrast to 12 days of the parental *N2* strain (Fig. 3B). In addition, the brood size of *viro-9(vir19*) animals was diminished by 36% compared to the parental *N2* (Fig. 3C). Results of these two assays suggested that VIRO-9 is important for the *C. elegans* lifespan and reproductive health, in the absence of virus infection.

### The *C. briggsae* ortholog *CBG23913* can functionally replace *C. elegans viro-9* during Orsay virus infection

**Fig 4:**
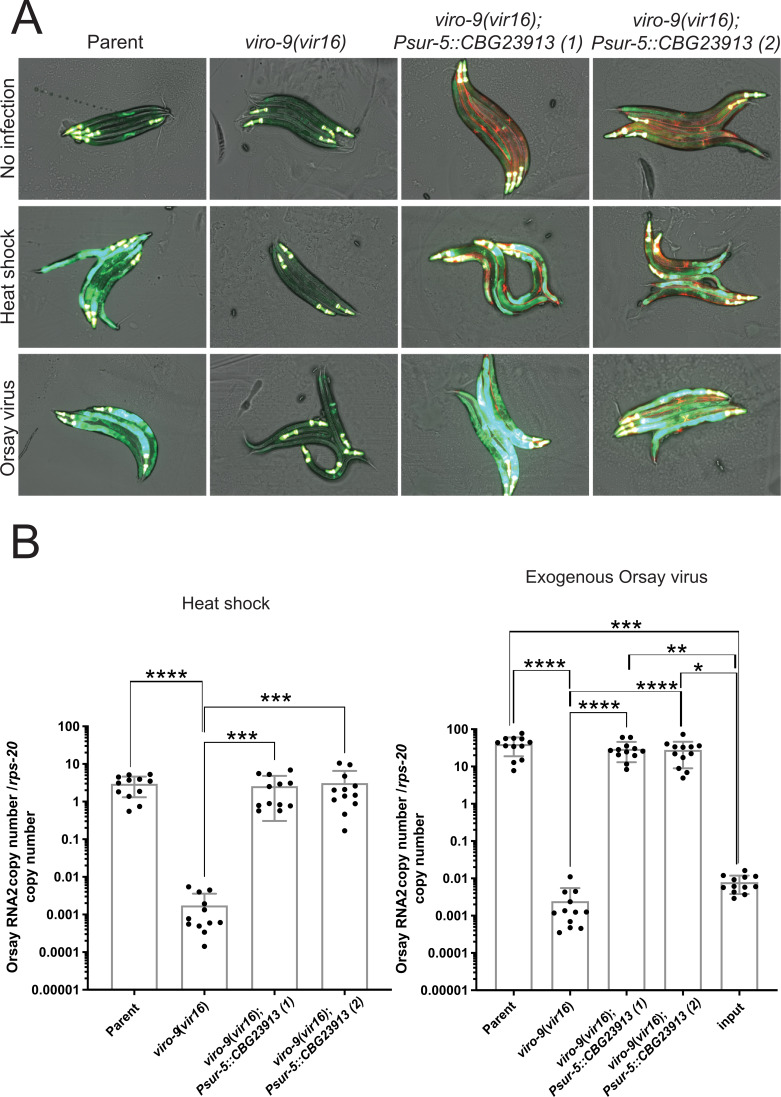
GFP expression and Orsay virus RNA2 levels in *C. elegans* animals complemented with *C. briggsae CBG23913*. (**A**) Animals that ectopically express the *CBG23913* show rescue in GFP expression upon Orsay virus infection. The *Pmyo-3::mCherry* was used as a fluorescent marker, indicated by the mCherry fluorescence in the body wall muscle of transgenic animals. (**B**) qRT-PCR quantification of Orsay virus load in *CBG23913*-transgenic animals upon exogenous Orsay virus infection or initiation of replication from the Orsay virus transgene by heat shock. The mean and SD of at least three independent experiments are represented. The Kruskal-Wallis test was performed, followed by Dunn’s multiple comparison test. Adjusted *P*-values are indicated as follows: *, *P* < 0.05; **, *P* < 0.01; ***, *P* < 0.001; ****, *P* < 0.0001. Non-significant *P*-values are not shown.

To test whether the function of VIRO-9 is evolutionarily conserved, we asked whether the orthologous gene in *C. briggsae*, a nematode species separated from *C. elegans* by 80–110 million years (19), can rescue Orsay virus infection in *C. elegans viro-9(vir16*) mutants. We ectopically expressed the *C. briggsae CBG23913* in *viro-9(vir16*). We obtained two independently generated transgenic lines and monitored GFP expression and virus load upon heat shock or exogenous Orsay virus infection. *CBG23913* overexpression rescued the loss of GFP expression upon heat shock and exogenous Orsay virus infection in the rescue lines (Fig. 4A), as well as the Orsay virus load (Fig. 4B). These results demonstrate that CBG23913 can functionally replace VIRO-9 in *C. elegans*. This supports that, at least in the context of virus infection, the functions of *C. elegans viro-9* and its *C. briggsae* ortholog are evolutionarily conserved.

### Amino acids important for virus infection are conserved across SRR1 domain-containing genes in the Animalia, Fungi, and Plantae kingdoms

**Fig 5:**
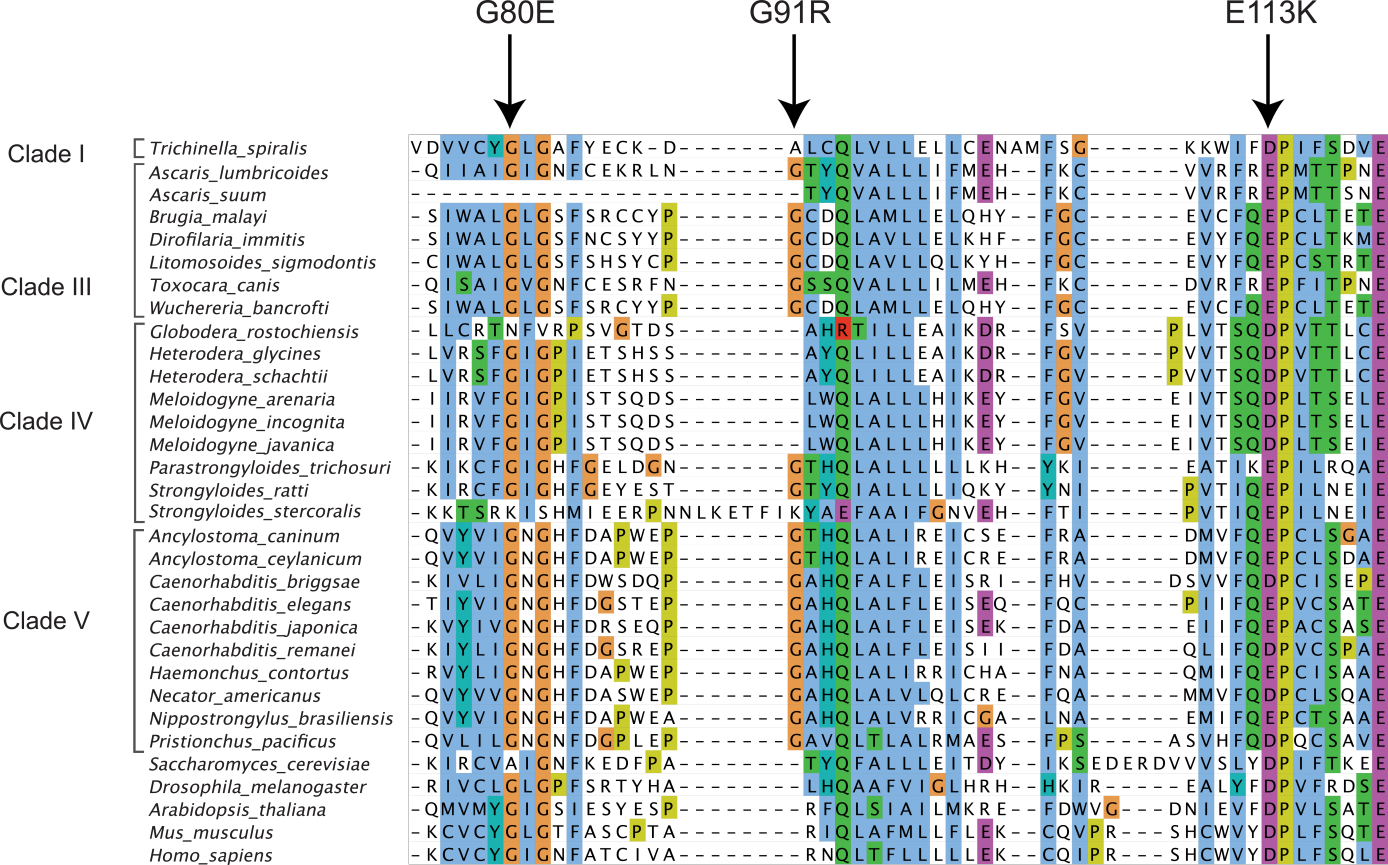
Alignment of SRR1 domain-containing genes from representative species from the Animalia, Fungi, and Plantae kingdoms. Nematode sequences representing different clades are indicated in half brackets. The bottom five rows show sequences corresponding to the SRR1 domain-containing genes of *Saccharomyces cerevisiae*, *Drosophila melanogaster*, *Arabidopsis thaliana*, *Mus musculus*, and *Homo sapiens*. Arrows indicate residue positions altered in mutant lines *viro-9(vir16)*, *viro-9(vir17)*, and *viro-9(vir18),* corresponding to missense mutations G80E, G91R, and E113K, respectively.

All three *viro-9* mutant alleles, *viro-9(vir16*) G80E, *viro-9(vir17*) G91R, and *viro-9(vir18*) E113K, had Orsay virus loads 15,000- to 39,000-fold lower than the unmutagenized parental line (Fig. 1B). This result demonstrated the importance of these amino acids for the function of VIRO-9 to promote Orsay virus replication. To determine if these amino acids are conserved in the SRR1 domain across multiple kingdoms, we compared SRR1 domain-containing sequences of nematodes, *Saccharomyces cerevisiae*, *Drosophila melanogaster*, *Arabidopsis thaliana*, *Mus musculus*, and *Homo sapiens* (Fig. 5) by multiple sequence alignment. Nematodes are taxonomically classified into five different clades, and we included species from each clade that are well represented in comparative studies (28). The number of nematodes included in the comparison ranged from 1 to 10, since information was limited for nematodes in clades I and II, whereas clade V, which includes *Caenorhabditis* nematodes, is well studied. We found that the G80 and E113 sites are highly conserved between nematodes, *Drosophila*, *Arabidopsis*, and mammals. The amino acid negative charge at the E113 site was conserved across all sequences included in the alignment. The conservation of G80 and E113 across multiple eukaryotic kingdoms suggests that these two residues are likely critical for one or more functions of the SRR1 domain-containing proteins in general. In contrast, G91 was present in clade III and V nematodes but absent in nematodes of other clades or other organisms outside of the nematode kingdom, suggesting that this residue is specifically important for a subset of nematodes containing SRR1 domain proteins.

To gain further insight into the potential role of the three residues in the Viro mutants, we used AlphaFold2 (29) to predict the structures of the wild-type and variant forms of VIRO-9 (Fig. S2). Within the wild-type structure, G80 and E113 fell within the junction between an alpha helix and a beta strand, exposed at the surface of VIRO-9. G91 is present at the edge of an alpha helix, facing outward toward the surface. None of the three loss-of-function point mutations dramatically altered the predicted overall VIRO-9 structure. These observations suggest that these non-conservative mutations (G->E, G->R, and E->K) likely impact interactions with other proteins or biomolecules.

## DISCUSSION

Prior genetic screens in *C. elegans* using Orsay virus identified numerous pro-viral host factors. These genes included *sid-3*, *viro-2*, *nck-1*, *drl-1*, and *hipr-1*, which are important for Orsay virus entry (7–9), and *alg-1*, which is important for the replication stage of the Orsay virus lifecycle (10). By modifying the genetic screening strategy and initiating the virus life cycle from an integrated transgene, we were able to focus the current screen on genes involved in virus replication, thereby excluding host factors involved in virus entry. This approach uncovered a novel gene, *viro-9,* involved in Orsay virus replication in *C. elegans*.

Because *viro-9* had not previously been characterized in *C. elegans*, we addressed the impact of the deletion of *viro-9* on *C. elegans* life history traits. We observed a moderate reduction in lifespan and brood size in *viro-9* mutants. While the mechanistic basis for these phenotypes is unknown, the few studies in other organisms of the SRR1 domain provide some possible explanations. The human SRR1 domain-containing protein SRRD has recently been described to play a role in regulating intermediate filaments, vimentin cage formation, and aggresome assembly, which are important for establishing protein turnover (21). It is possible that disturbance to the actin cytoskeletal network could reduce the brood size by compromising the germline structural integrity (30, 31). Also, disruption of protein homeostasis has previously been associated with a reduction in brood size (32, 33).

In terms of the mechanism by which *viro-9* impacts Orsay virus replication, one possibility is that VIRO-9 could, analogously to human SRRD, regulate intermediate filaments and intestinal cell cytoskeletal structure. Disturbances to the actin cytoskeleton have been reported to impact Orsay virus infection (34). As another possibility, VIRO-9 could interact with other known pathways that promote Orsay virus replication. The only other *C. elegans* gene reported to date that is important for the replication step of the Orsay virus lifecycle is *alg-1* (10). ALG-1 is a key component of the RNA-induced silencing complex (RISC) in processing host microRNAs (35, 36). While the mechanism of ALG-1 during Orsay virus infection remains to be defined, it is possible that its role in small RNA processing is important based on the observation that AIN-1, a known protein interactor of ALG-1 also found in the RISC, is also needed for Orsay virus infection (10). While there is no data linking *alg-1* and *viro-9* together, the mutation of these genes leads to similar Orsay virus phenotypes. Structural prediction with AlphaFold2 found that the three loss-of-function amino acid mutation sites were all surface exposed (Fig. S2). The changes from neutral charge to a charged and bulkier amino acid residue in G80E and G91R and the charge reversal of E113K could lead to disruption of important protein-protein interactions. As another explanation for the low virus replication level, it is possible that, since the VIRO-9 mutations have modest impacts on lifespan and progeny production, the reduction in general animal health indirectly impacts Orsay virus infection. Given the modest impacts, though, on lifespan and progeny, as compared to the >1,000-fold impact on Orsay virus RNA levels, we think this is unlikely. Further research is needed to determine whether VIRO-9 is directly involved in virus replication.

To gain insights into the conservation of the function of genes containing the SRR1 domain, we expressed *CBG23913*, the *C. briggsae* ortholog of *viro-9*, in *viro-9(vir16*) animals and observed rescued GFP expression and virus replication levels (Fig. 4). The ability of *CBG23913* to rescue the virus replication defect implies that the function of the SRR1 domain-containing genes in *C. elegans* and *C. briggsae* is evolutionary conserved across 80–110 million years (19, 37). To provide a basis for comparison, the length of separation between *C. briggsae* and *C. elegans* is comparable to the evolutionary distance between mice and humans (37). Furthermore, alignment of the SRR1 domain-containing sequences revealed that the G80 and E113 residues that have been altered in the *viro-9* mutants are also present in SRR1 domain-containing genes of organisms in multiple other kingdoms. Whether these residues are only important for promoting virus replication or if they are important for the other reported roles of the SRR1 domain has yet to be determined. For example, it will be important in future studies to assess whether disrupting the G80 and E113 residues in the SRR1 domain contributes to a disruption in red light sensing in Arabidopsis (18), disorganized microtubule organization in yeast (16), or cell proliferation defects in mammalian cells (20). In the context of mammalian virus infection, the SRR1 domain-containing gene SRRD has appeared as a pro-viral hit in previously performed CRISPR/Cas9 screens in Huh7.5.1, human cells, with hepatitis C virus (23) and Vero E6, African green monkey cells, with SARS-CoV-2 (24, 25). Together with our data showing that diverse SRR1 domain proteins in both *C. elegans* and *C. briggsae* are important for Orsay virus infection, these results suggest that SRR1 domain-containing proteins play key roles in promoting infection of viruses in multiple families that infect hosts ranging from nematodes to mammals.

Overall, our unbiased screen uncovered a novel pro-viral gene, *viro-9*, which further extends our knowledge of host factors important in *C. elegans* for Orsay virus replication. Moreover, this defines unambiguously for the first time a pro-viral function of the SRR1 domain. Beyond explicitly defining the conservation of the pro-viral function of the *C. briggsae* ortholog, the extensive conservation of the G80 and E113 residues in SRR1 domain proteins in multiple kingdoms strongly suggests SRR1 domains likely play conserved proviral functions in additional organisms. These findings highlight the strengths of the *C. elegans-*Orsay virus infection system in understanding fundamentally important genes and protein domains involved in host-virus interaction.

## MATERIALS AND METHODS

### *C. elegans* strains and culturing

*C. elegans* strains *viro-9(sy1174) (PS8012*) and *rde-1(ne219) (WM27*) were obtained from the CGC. *C. elegans* animals were grown on a lawn on *E. coli* OP50 on nematode growth medium (NGM) plates. Animals were kept in a 20°C incubator and maintained by propagating progeny on new NGM plates every 3–4 days. A full list of strains used for experiments is included in Table S1.

### Orsay virus infection

Orsay virus stocks were prepared from a large-scale liquid culture and passed through a 0.22 µm filter (7). One hundred-microliter aliquots were stored at −80°C. To prepare a synchronized *C. elegans* population for infection, a mixed population of *C. elegans* animals was expanded on a 10 cm plate and bleached in a 15 mL conical tube. Bleached embryos were recovered in M9 buffer for 16 hours at room temperature with constant rotation. For each experimental replicate, 500 L1 larvae were placed in one well of a six-well plate and allowed to recover at 20°C. For the infection, the stock virus was diluted at a 1:10 ratio in M9 buffer, and 20 µL of the diluted virus was added to the synchronized population of *C. elegans* animals. Three days after infection, animals were collected in 1 mL of M9 buffer, followed by centrifugation at 2,000 rpm for 1 minute and removal of the supernatant. The pellet of animals was resuspended in 300 µL of Trizol and snap-frozen in liquid nitrogen. The Direct-zol RNA Miniprep (Zymo Research) kit was used to extract the RNA, followed by quantification of RNA by qRT-PCR. To establish a baseline, we created a control condition in which we added the virus to a well containing only the bacterial lawn. Three days later, we combined the residual virus together with uninfected *C. elegans* animals for qRT-PCR. For initiating the virus life cycle intracellularly, WUM29 animals were synchronized by bleaching and recovered at 20°C until they reached the L4/young adult stage. Animals were incubated at 33°C for 2 hours for the acute heat shock phase, followed by recovery at 23°C for 48 hours. At least three independent experiments were performed, where each experiment was composed of three replicates per experimental condition.

### *N. parisii* infection

*N. parisii* stocks were prepared as described previously (11). Briefly, *N. parisii* culture was propagated on a large-scale *C. elegans* population, followed by homogenization and filtration to remove *C. elegans* debris. For infection experiments, *C. elegans* animals were bleach-synchronized as described above and allowed to recover at 20°C. Twenty microliters of the *N. parisii* stock was used to infect a synchronized population of *C. elegans* animals at the L2 stage and monitored for 3 days following infection for GFP expression.

### Orsay virus RNA quantification

Extracted RNA was diluted 1:100 for qRT-PCR quantification. Using the TaqMan Fast Virus 1-Step Master Mix (ThermoFisher), Orsay virus RNA2 was amplified with primers GW303 and GW304. *rps20*, which comprises the small ribosomal subunit, was quantified as an internal control host transcript. The copy numbers of RNA2 or *rps20* were obtained by interpolating a standard curve generated from *in vitro* transcripts of RNA2 or *rps20*.

### Microscopy image acquisition of GFP-expressing *C. elegans* animals

Animals were paralyzed with 40 mM levamisole and staged on a 3% agarose pad of 1 mm thickness. Images were acquired using the Zeiss Axio Imager D1 inverted fluorescence microscope (Carl Zeiss Inc., Thornwood, NY), equipped with an EC Plan-Neofluar (NA 0.3) 10× objective (Zeiss) and an Axiocam 503 color camera (Zeiss). The X-Cite 120 mini LED light source (Excelitis Technologies, Waltham, MA) was used for capturing brightfield images, with an exposure time of 20 ms. For detecting GFP or mCherry, a GFP filter (ex 450–490 nm and em 500–550 nm) and a Cy3 (mCherry) filter (ex 537–563 and em 570–640 nm) were used. Exposure times were set to 300 ms and 150 ms for GFP and mCherry, respectively. Images were processed with the ZEN 2 (blue version) software.

### Forward genetic screen for mutants that fail to activate GFP expression following induction of the transgenic Orsay virus

The standard EMS protocol was followed as described previously (7), with modifications described below. Briefly, the *virIs1; jyIs8; rde-1* (WUM29) reporter strain was expanded on a 10 cm plate. Animals were subjected to mutagenesis by incubating in 50 mM EMS for 4 hours at 20°C. L4 animals were transferred onto a new 10 cm plate and allowed to expand to the F1 generation. Adult stage F1 animals were synchronized to obtain the F2 generation. F2 animals were allowed to recover at 20°C to reach the L4/young adult stage. At the late L4 stage, animals were heat shocked in an incubator set at 33°C for 2 hours, followed by recovery at 23°C for 48 hours. During the 48 hours, animals were monitored for GFP expression. Any GFP-off animals were isolated into individual plates and expanded separately for further rounds of confirmatory infection experiments with Orsay virus or *N. parisii*. For exogenous Orsay virus infection, synchronized animals were infected with Orsay virus as described under “Orsay virus infection.”

### Genetic mapping of candidate mutant strains

For whole-genome sequencing, candidate mutant lines were expanded on a 10 cm plate and lysed for DNA extraction with the DNeasy Blood and Tissue Kit (QIAGEN). For the F2 bulk segregation analysis, the *viro-16(vir16*) was used. At first, the *viro-9(vir16*) was crossed to the parent *virIs1; jyIs8; rde1* (WUM29), and the progeny was expanded to the F1 generation. F1 animals were isolated into single wells, and the F2 generation was infected with the Orsay virus. GFP-off F2 animals were singled out into individual wells and expanded further into duplicate wells of the F3 generation. One of the duplicate wells was infected with the Orsay virus, while the second well was set aside for DNA extraction. Orsay virus-infected wells were monitored for GFP expression and virus load. Uninfected wells corresponding to GFP-off and virus load-low phenotypes upon Orsay virus infection were pooled for DNA extraction using the DNeasy Blood and Tissue Kit (QIAGEN). For whole-genome sequencing samples or F2 bulk segregation analysis samples, the extracted DNA was used for library preparation using the Nextera library preparation kit (New England Biolabs). Raw sequencing reads were processed and analyzed using MiModD (http://mimodd.readthedocs.io/en/latest/; DOI 10.5281/zenodo.1189838).

### Transgene rescue

For creating the *viro-9* overexpression construct (*Psur-5::viro-9*), the *N2* genomic DNA was used as a template for amplifying the *viro-9* sequence using CF010_SacII_+21F_y55f3bl4g and CF011_BmtI_+1021R_y55f3bl4g. The amplified DNA was digested with SacII and BmtI, followed by ligation into an overexpression vector driven by the *sur-5* promoter. The *C. briggsae* ortholog of *viro-9* (*CBG23913*) was amplified using primers CF275_cbrY55_1F_SacII and CF276_cbrY55_1753R_BmtI off of DNA extracted from the *C. briggsae* reference strain AF16. The PCR product was digested with SacII and BmtI and inserted into the *sur-5* vector (*Psur-5::CBG23913*). The rescue constructs were injected into *viro-9(vir16*) at 5 ng/µL, together with a co-injection marker, *Pmyo-3::mCherry* (5 ng/µL) and 1 kb Plus DNA Ladder (New England Biolabs [NEB], N3200S; 100 ng/µL). Two independent transgenic lines with a transgene transmission rate of 25%–30% were used for experiments.

### CRISPR/Cas9 editing of *C. elegans* animals

A *C. elegans* line with a deletion of the full *viro-9* locus was generated using CRISPR/Cas9 technology as described previously, using the *dpy-10* co-conversion strategy (38, 39). Two crRNAs (CF127_CD.Cas9.RLFP3512.AA, CF128_CD.Cas9.BJHY7990.AG) were designed for targeting the *viro-9* locus using the Alt-R Custom Cas9 crRNA Design Tool available through Integrated DNA Technologies. A repair template (CF129_repair_−335_ 1428_Y55F3BL4) was designed, flanking 35 base pairs upstream and downstream of the crRNA target sites. The injection mix was prepared by combining the Cas9 protein (15 µM), tracrRNA (42 µM), dpy-10 crRNA (15 µM), dpy-10 repair template (0.5 µM), *viro-9* crRNAs (15 µM each), and *viro-9* repair template (0.5 µM). All reagents were obtained from IDT. The progeny of injected animals exhibiting the *dpy-10* phenotype were expanded and used for PCR verification of CRISPR/Cas9 editing at the *viro-9* locus.

### Characterization of life history traits

For the lifespan assay, 10 young adults were transferred into a well of a six-well plate and re-transferred to new wells every other day. Dead animals were recorded and removed from the wells. Any animals that crawled out of the wells were censored. To determine the brood size, 10 synchronized young adult animals were transferred into a well of a 6 cm plate. All animals were transferred to a new well daily, and the progeny was allowed to develop to the L4 stage in each well, prior to counting.

### Alignment of SRR1 domain-containing sequences

For previously characterized nematode species (28), the corresponding *viro-9* homolog amino acid sequences were obtained from BioMart (40). Other SRR1 domain-containing sequences were obtained from NCBI for *Saccharomyces cerevisiae* (NP_013516.1), *Drosophila melanogaster* (NP_650691.1), *Arabidopsis thaliana* (NP_200764.1), *Mus musculus* (NP_001346317.1), and *Homo sapiens* (NP_001013716.2). Sequences were aligned using Clustal Omega (41) and visualized using Jalview (42). Within Jalview, residues were assigned a color if they met the minimum requirements based on the Clustal X criteria. Briefly, amino acid residues in the hydrophobic, positive charge, negative charge, polar, and aromatic categories were colored in blue, red, magenta, green, and cyan, respectively. Cysteine, glycine, and proline were colored in pink, orange, and yellow, respectively. No color was assigned if the amino acid at a particular position did not meet the minimum criteria.

### Structure prediction of wild-type and mutant VIRO-9

Structures of wild-type and mutant VIRO-9 were predicted using AlphaFold2 (29). The output PDB files were visualized and annotated using UCSF Chimera (43). Predicted structures of the wild-type and mutant VIRO-9 were colored in beige and blue, respectively. An overlay of the wild-type and mutant structures was created in UCSF Chimera.

### Statistical testing

At least three independent experiments were performed. GraphPad Prism 8.0.2 was used for statistical testing. Three independent experiments were performed for Orsay virus infection experiments, and the Kruskal-Wallis test was performed for comparing multiple groups. Tests for multiple comparisons were performed using Dunn’s test. Adjusted *P*-values were reported in the text and figures. The log-rank test and the Mann-Whitney test were used for determining the statistical significance of the median survival times and brood sizes, respectively. Statistical significance is indicated as follows: ns (not significant), *, *P* < 0.05; **, *P* < 0.01; ***, *P* < 0.001; ****, *P* < 0.0001.

## Data Availability

Raw sequencing reads of the EMS mutant *C. elegans* lines *viro-9*(*vir16*), *viro-9*(*vir17*), *viro-9*(*vir18*), and the unmutagenized parent were deposited to NCBI under BioProject accession PRJNA1235832.

## SUPPLEMENTAL MATERIAL

Figure S1 jvi.00521-25-s0001.tiff qRT-PCR quantification of Orsay virus RNA2.

Figure S2 jvi.00521-25-s0002.tiff AlphaFold2-predicted structures of VIRO-9.

Tables S1 to S3 jvi.00521-25-s0003.pdf Table S1, strains; Table S2, oligonucleotides and probes; Table S3, qPCR values.

ASM does not own the copyrights to Supplemental Material that may be linked to, or accessed through, an article. The authors have granted ASM a non-exclusive, world-wide license to publish the Supplemental Material files. Please contact the corresponding author directly for reuse.
