# Supplementary figures and images for: An SRR1 domain-containing protein is required for efficient Orsay virus replication in Caenorhabditis elegans
Source: J Virol. 2025 Sep 3;99(9):e00521-25. doi: 10.1128/jvi.00521-25 (PMC12455975; doi:10.1128/jvi.00521-25)

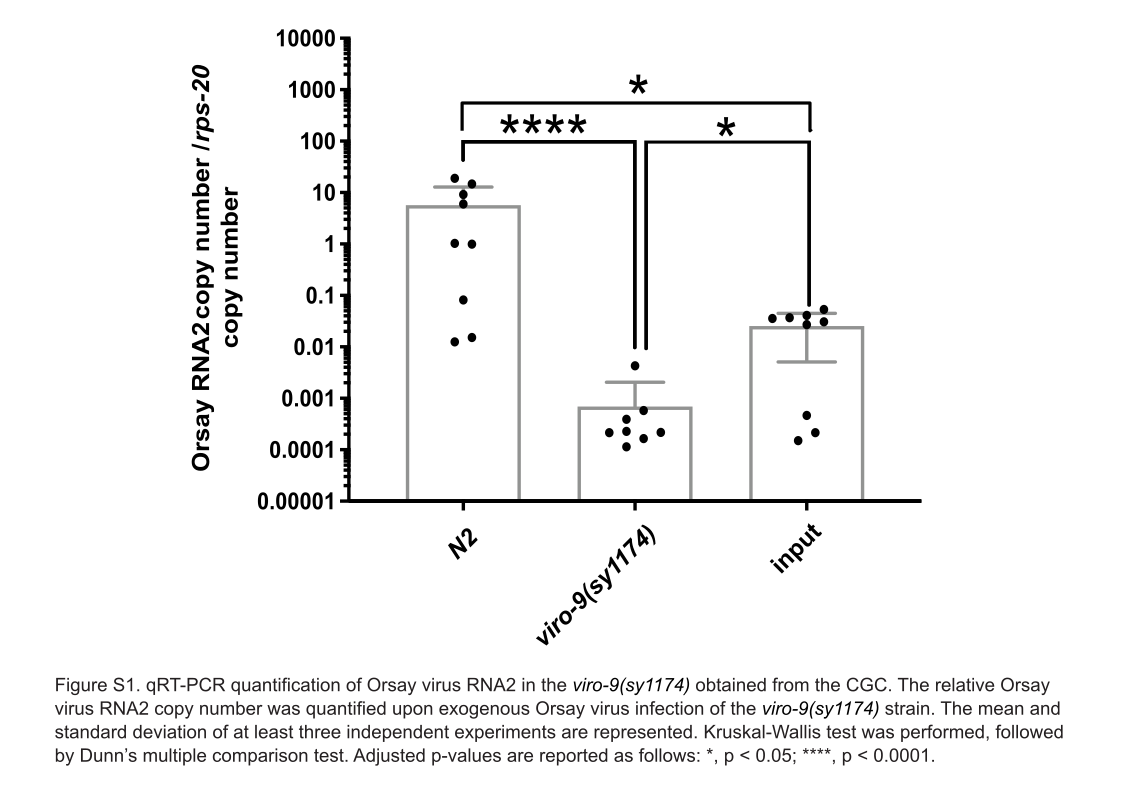

Supplement: Figure S1 — qRT-PCR quantification of Orsay virus RNA2. [file jvi.00521-25-s0001.tiff]

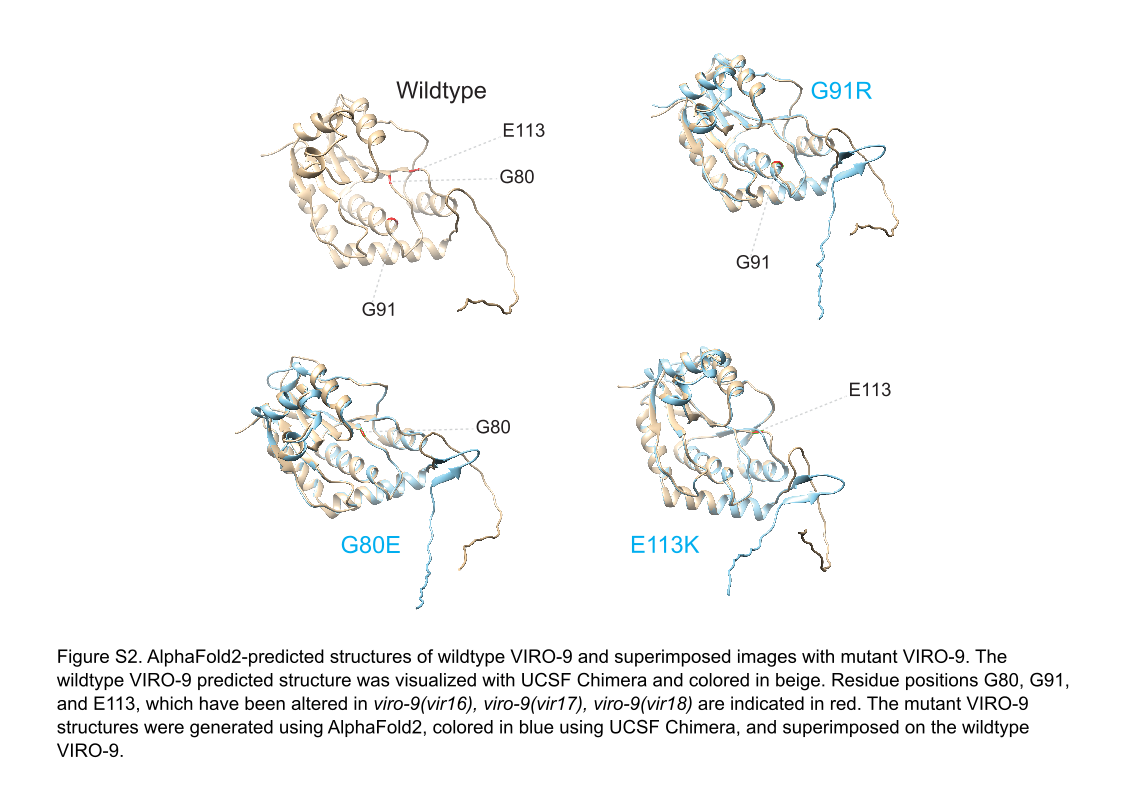

Supplement: Figure S2 — AlphaFold2-predicted structures of VIRO-9. [file jvi.00521-25-s0002.tiff]
