# Supplementary material for: An SRR1 domain-containing protein is required for efficient Orsay virus replication in Caenorhabditis elegans
Source: J Virol. 2025 Sep 3;99(9):e00521-25. doi: 10.1128/jvi.00521-25 (PMC12455975; doi:10.1128/jvi.00521-25)
Supplement: Tables S1 to S3 — Table S1, strains; Table S2, oligonucleotides and probes; Table S3, qPCR values. [file jvi.00521-25-s0003.pdf]

Table S1. Strains used in this study

| Laboratory name | Strain name | Relevant genotype | Source | Notes |
|---|---|---|---|---|
| N2 | *N2* | | CGC | |
| WM27 | *rde-1(ne219)* | *[rde-1(ne219)V]* | Lab strain | |
| WUM31 | *jyls8; rde-1 (ne219)* | *{jyIs8[Ppals-5::GFP; Pmyo-2::mCherry]; rde-1 (ne219) V}* | Lab strain | |
| WUM29 | *virIs1; jyIs8; rde1 (ne219)* | *{virIs1[PHIP::OrsayRNA1;PHIP::OrsayRNA2; Pmyo-2::YFP]; jyIs8[Ppals- 5::GFP; Pmyo-2::mCherry]; rde-1(ne219) V}* | Lab strain | |
| WUM120 | *viro-9(vir16)* | *{virIs1[PHIP::OrsayRNA1;PHIP::OrsayRNA2; Pmyo-2::YFP]; jyIs8[Ppals-5::GFP; Pmyo-2::mCherry]; rde-1(ne219) V; viro-9(vir16) IV}* | Lab strain | |
| WUM121 | *viro-9(vir17)* | *{virIs1[PHIP::OrsayRNA1;PHIP::OrsayRNA2; Pmyo-2::YFP]; jyIs8[Ppals-5::GFP; Pmyo-2::mCherry]; rde-1(ne219) V; viro-9(vir17) IV}* | Lab strain | |
| WUM122 | *viro-9(vir18)* | *{virIs1[PHIP::OrsayRNA1;PHIP::OrsayRNA2; Pmyo-2::YFP]; jyIs8[Ppals-5::GFP; Pmyo-2::mCherry]; rde-1(ne219) V; viro-9(vir18) IV}* | Lab strain | |
| WUM123 | *viro-9(vir16); virEx80[Psur-5::viro-9]* | *{virIs1[PHIP::OrsayRNA1;PHIP::OrsayRNA2; Pmyo-2::YFP]; jyIs8[Ppals-5::GFP; Pmyo-2::mCherry]; rde-1(ne219) V; viro-9(vir16) IV virEx80[Psur-5::viro-9;Pmyo-3::mCherry]}* | Lab strain | |
| WUM124 | *viro-9(vir16); virEx81[Psur-5::viro-9]* | *{virIs1[PHIP::OrsayRNA1;PHIP::OrsayRNA2; Pmyo-2::YFP]; jyIs8[Ppals-5::GFP; Pmyo-2::mCherry]; rde-1(ne219) V; viro-9(vir16) IV virEx81[Psur-5::viro-9;Pmyo-3::mCherry]}* | Lab strain | |
| WUM125 | *viro-9(vir16); virEx82[Psur-5::CBG23913]* | *{virIs1[PHIP::OrsayRNA1;PHIP::OrsayRNA2; Pmyo-2::YFP]; jyIs8[Ppals-5::GFP; Pmyo-2::mCherry]; rde-1(ne219) V; viro-9(vir16) IV virEx82[Psur-5::CBG23913;Pmyo-3::mCherry]}* | Lab strain | |
| WUM126 | *viro-9(vir16); virEx83[Psur-5::CBG23913]* | *{virIs1[PHIP::OrsayRNA1;PHIP::OrsayRNA2; Pmyo-2::YFP]; jyIs8[Ppals-5::GFP; Pmyo-2::mCherry]; rde-1(ne219) V; viro-9(vir16) IV virEx83[Psur-5::CBG23913;Pmyo-3::mCherry]}* | Lab strain | |
| WUM127 | *viro-9(vir19)* | *[viro-9(vir19) IV]* | Lab strain | Full deletion of the *viro-9* locus |
| PS8012 | *Y55F3BL.4(sy1174)* | *[viro-9(sy1174) IV]* | CGC | |

Table S2. Oligos and probes used in this study.

| Primer Name | Sequence | Notes |
|---|---|---|
| GW303 | CCGGCGACAATGTGTACCA | |
| GW304 | CCAGCCCTCCGTTGACAA | |
| Orsay_RNA2 Probe | /56-FAM/CGAGGCCACCCATATCAGGGCCTA/36-TAMSp/ | |
| GW0314 | TGGATCCAACGCCGTTAAC | |
| GW0315 | CGATTTGCAGTGGCTTGCT | |
| Orsay_RNA1 Probe | /56-FAM/TGGACCACTGAGCAAGTGATCGAGAACC/36-TAMSp/ | |
| KC291 | TTCCAGATGCGCATCCAC | |
| KC292 | GGCTCTGGTGACCTCAAT | |
| RPS-20_e3-4 Probe | /56-FAM/TCGATCTCC/ZEN/ATCGAGCCAGGAGTC/3IABkFQ/ | |
| CF010_SacII_+21F_y55f3bl4g | ATATccgcggATGGAAGACGACGGATTCACCC | |
| CF011_BmtI_+1021R_y55f3bl4g | ATATgctagcTTATTGATTATCACAAGAAGAC | |
| CF275_cbrY55_1F_SacII | ATATccgcggATGGAAGACGATGGATTCACATTGG | |
| CF276_cbrY55_1753R_BmtI | ATATgctagcTTATTCAAAATCGGGACACATGATTACAGTAC | |
| CF127_CD.Cas9.RLFP3512.AA | caagtcgggagcctggtttt | crRNA1 for generating *viro-9* full knockout |
| CF128_CD.Cas9.BJHY7990.AG | caaccagacgtcagaagtac | crRNA2 for generating *viro-9* full knockout |
| CF129_repair_-335_ 1428_Y55F3BL4 | aaaaaattactgaaatttcaagtcgggagcctggtccagacgtcagaagtacggaaattggctgaaaaac | Repair template |
| CF092_-374F_Y55F3BL4 | caattttcgaaatttttctgtggg | CF092/CF164: del PCR |
| CF164_Y55F3BL4_1492R | accaccatatcggagtgtcatttg | |
| CF080_+219R_Y55F3BL.4g | CCAATCCGCTTTTCAGAATCCC | CF092/CF080: WT PCR |

Table S3. Raw results of qRT-PCR assays.

Figure 1B. Quantification of Orsay virus RNA2 upon exogenous Orsay virus infection or initiating the virus life cycle by heat shock.

Heat shock

| Parent | | | | |
|---|---|---|---|---|
| RNA2 CT | rps-20 CT | RNA2 copy number | rps-20 copy number | Orsay virus RNA2/rps-20 copy number ratio |
| 19.499 | 21.214 | 122210.624 | 110851.409 | 1.10247245 |
| 18.503 | 21.769 | 117916.170 | 74268.300 | 1.58770524 |
| 28.247 | 21.014 | 233779.901 | 128010.356 | 1.82625772 |
| 18.574 | 22.608 | 88154.442 | 40577.194 | 2.17251203 |
| 19.223 | 21.072 | 222557.831 | 122715.314 | 1.81361090 |
| 26.592 | 19.823 | 142479.815 | 302062.332 | 0.47169011 |
| 18.772 | 22.764 | 218324.520 | 36273.318 | 6.01887377 |
| 18.697 | 20.280 | 194220.385 | 217200.416 | 0.89419895 |
| 30.516 | 21.425 | 204575.294 | 95149.735 | 2.15003534 |
| Viro-9 | | | | |
| RNA2 CT | rps-20 CT | RNA2 copy number | rps-20 copy number | Orsay virus RNA2/rps-20 copy number ratio |
| 26.955 | 20.154 | 289.101 | 237812.705 | 0.00121567 |
| 29.148 | 19.581 | 702.403 | 359593.165 | 0.00195333 |
| 29.403 | 21.154 | 155.720 | 115749.199 | 0.00134533 |
| 27.982 | 20.253 | 901.320 | 221540.862 | 0.00406842 |
| 29.408 | 19.153 | 346.788 | 489584.780 | 0.00070833 |
| 30.077 | 22.141 | 130.252 | 56800.698 | 0.00229314 |
| 28.355 | 24.246 | 60.803 | 12458.221 | 0.00488059 |
| 29.478 | 21.628 | 268.389 | 82245.260 | 0.00326327 |
| 35.089 | 23.450 | 124.116 | 22110.633 | 0.00561341 |
| Viro-10 | | | | |
| RNA2 CT | rps-20 CT | RNA2 copy number | rps-20 copy number | Orsay virus RNA2/rps-20 copy number ratio |
| 29.686 | 19.737 | 130.672 | 321234.203 | 0.00040678 |
| 32.002 | 20.329 | 107.569 | 209709.787 | 0.00051294 |
| 27.437 | 22.897 | 21.903 | 32938.536 | 0.00066497 |
| 27.153 | 20.555 | 82.228 | 178205.462 | 0.00046142 |
| 25.486 | 20.389 | 613.121 | 200894.702 | 0.00305195 |
| 28.997 | 18.814 | 1927.107 | 624971.352 | 0.00308351 |
| 28.410 | 28.045 | 2.628 | 806.493 | 0.00325804 |

| | | | | |
|---|---|---|---|---|
| 29.831 | 21.875 | 258.562 | 68798.467 | 0.00375826 |
| 27.427 | 24.390 | 97.340 | 11234.940 | 0.00866406 |
| Viro-11 | | | | |
| RNA2 CT | rps-20 CT | RNA2 copy number | rps-20 copy number | Orsay virus RNA2/rps-20 copy number ratio |
| 27.769 | 21.519 | 504.277 | 88949.710 | 0.00566923 |
| 28.923 | 20.674 | 401.421 | 163488.367 | 0.00245535 |
| 27.386 | 20.525 | 181.741 | 182132.316 | 0.00099785 |
| 28.105 | 20.553 | 172.747 | 178393.942 | 0.00096835 |
| 26.771 | 22.127 | 318.823 | 57400.139 | 0.00555440 |
| 26.476 | 21.347 | 797.093 | 100677.739 | 0.00791727 |
| 29.775 | 21.552 | 507.756 | 86835.930 | 0.00584730 |
| 28.031 | 23.984 | 101.173 | 15054.620 | 0.00672043 |
| 26.748 | 22.246 | 335.390 | 52683.425 | 0.00636615 |

Exogenous Orsay virus

| Parent | | | | |
|---|---|---|---|---|
| RNA2 CT | rps-20 CT | RNA2 copy number | rps-20 copy number | Orsay virus RNA2/rps-20 copy number ratio |
| 15.221 | 22.439 | 2228125.090 | 45824.950 | 48.62253194 |
| 14.317 | 22.529 | 4146276.406 | 42963.161 | 96.50771227 |
| 14.362 | 22.342 | 4020323.143 | 49149.966 | 81.79706870 |
| 14.241 | 22.239 | 4369786.517 | 52956.869 | 82.51595344 |
| 15.226 | 22.417 | 2221417.726 | 46554.803 | 47.71618824 |
| 13.741 | 22.165 | 6162658.111 | 55827.504 | 110.38749109 |
| 17.573 | 17.776 | 1739530.333 | 1320582.320 | 1.31724491 |
| 14.057 | 21.514 | 442919.074 | 89301.083 | 4.95983988 |
| 25.979 | 18.266 | 4958504.850 | 927630.934 | 5.34534228 |
| 11.462 | 20.956 | 5473482.420 | 133491.111 | 41.00259834 |
| 14.960 | 18.720 | 29484348.559 | 668794.518 | 44.08581079 |
| 24.347 | 20.405 | 2666116.197 | 198485.430 | 13.43230176 |
| Viro-9 (14-0-2) | | | | |
| RNA2 CT | rps-20 CT | RNA2 copy number | rps-20 copy number | Orsay virus RNA2/rps-20 copy number ratio |
| 29.034 | 21.698 | 168.345 | 78160.823 | 0.00215383 |
| 28.608 | 20.976 | 225.572 | 131532.968 | 0.00171494 |
| 28.687 | 21.219 | 213.716 | 110444.235 | 0.00193506 |
| 27.427 | 23.237 | 224.281 | 25782.406 | 0.00869899 |

| | | | | |
|---|---|---|---|---|
| 25.733 | 20.131 | 508.015 | 241871.830 | 0.00210035 |
| 28.238 | 21.257 | 1626.986 | 107401.627 | 0.01514862 |
| 27.105 | 24.624 | 26.538 | 9491.678 | 0.00279589 |
| 27.120 | 19.781 | 633.653 | 311312.455 | 0.00203543 |
| 26.257 | 19.837 | 627.060 | 298882.360 | 0.00209802 |
| Viro-10 (28-0-4) | | | | |
| RNA2 CT | rps-20 CT | RNA2 copy number | rps-20 copy number | Orsay virus RNA2/rps-20 copy number ratio |
| 28.420 | 21.399 | 256.656 | 96982.221 | 0.00264642 |
| 28.499 | 22.200 | 243.192 | 54463.086 | 0.00446526 |
| 28.990 | 21.765 | 173.525 | 74496.520 | 0.00232931 |
| 28.699 | 17.515 | 1135.106 | 1593599.821 | 0.00071229 |
| 27.510 | 21.243 | 211.977 | 108509.870 | 0.00195353 |
| 27.518 | 16.561 | 479.828 | 3170334.509 | 0.00015135 |
| 26.639 | 25.396 | 290.828 | 5439.416 | 0.05346674 |
| 33.303 | 21.393 | 872.718 | 97415.662 | 0.00895871 |
| 28.594 | 27.830 | 8.959 | 941.365 | 0.00951688 |
| Viro-11 (30-0-1) | | | | |
| RNA2 CT | rps-20 CT | RNA2 copy number | rps-20 copy number | Orsay virus RNA2/rps-20 copy number ratio |
| 28.518 | 22.163 | 55904.914 | 240.007 | 0.00429313 |
| 28.840 | 21.756 | 74961.041 | 192.316 | 0.00256555 |
| 28.212 | 21.822 | 71481.230 | 296.142 | 0.00414293 |
| 26.990 | 18.598 | 477.016 | 730135.156 | 0.00065333 |
| 26.288 | 19.628 | 685.758 | 347486.408 | 0.00197348 |
| 13.913 | 18.416 | 1110.551 | 832640.926 | 0.00133377 |
| 28.915 | 21.203 | 227.740 | 111675.790 | 0.00203930 |
| 27.683 | 20.975 | 182.679 | 131692.540 | 0.00138716 |
| 19.447 | 21.337 | 426.084 | 101430.922 | 0.00420073 |
| Input | | | | |
| RNA2 CT | rps-20 CT | RNA2 copy number | rps-20 copy number | Orsay virus RNA2/rps-20 copy number ratio |
| 26.614 | 22.975 | 887.851 | 31157.521 | 0.02849557 |
| 26.801 | 24.019 | 780.696 | 14680.127 | 0.05318045 |
| 26.601 | 23.270 | 896.164 | 25181.659 | 0.03558796 |
| 27.087 | 22.577 | 641.459 | 41489.068 | 0.01546093 |
| 27.287 | 23.402 | 559.286 | 22895.870 | 0.02442739 |
| 26.773 | 22.693 | 796.257 | 38171.837 | 0.02085980 |
| 25.958 | 17.716 | 1373.936 | 1378856.193 | 0.00099643 |

| | | | | |
|---|---|---|---|---|
| 24.692 | 20.260 | 1393.866 | 220393.297 | 0.00632445 |
| 31.723 | 18.430 | 3325.673 | 823995.327 | 0.00403603 |
| 25.116 | 19.695 | 4214.609 | 331280.389 | 0.01272218 |
| 25.334 | 20.968 | 2485.568 | 132301.115 | 0.01878720 |
| 28.617 | 19.258 | 2139.567 | 453666.085 | 0.00471617 |

Figure 2C. qRT-PCR quantification of Orsay virus RNA2 upon exogenous Orsay virus infection or the initiation of the Orsay virus life cycle by heat shock.

Heat shock

| Parent | | | | |
|---|---|---|---|---|
| RNA2 CT | rps-20 CT | RNA2 copy number | rps-20 copy number | Orsay virus RNA2/rps-20 copy number ratio |
| 19.480 | 22.784 | 119404.978 | 35737.010 | 3.34121347 |
| 14.030 | 19.976 | 5051686.744 | 270458.308 | 18.67824576 |
| 15.169 | 20.361 | 2310266.770 | 204886.768 | 11.27582220 |
| 17.184 | 20.545 | 578533.544 | 179492.249 | 3.22316728 |
| 17.395 | 20.708 | 500586.662 | 159525.401 | 3.13797464 |
| 17.618 | 20.291 | 429257.820 | 215542.831 | 1.99151982 |
| 15.999 | 20.348 | 206905.341 | 1306077.089 | 6.31243777 |
| 15.198 | 20.258 | 220736.516 | 2263525.228 | 10.25442127 |
| 15.207 | 21.084 | 121734.950 | 2250052.045 | 18.48320511 |
| 16.527 | 20.835 | 908290.326 | 145626.738 | 6.23711234 |
| 15.472 | 20.219 | 1876093.508 | 226967.665 | 8.26590656 |
| 18.079 | 22.259 | 312826.182 | 52178.123 | 5.99535140 |
| viro-9(vir16) | | | | |
| RNA2 CT | rps-20 CT | RNA2 copy number | rps-20 copy number | Orsay virus RNA2/rps-20 copy number ratio |
| 30.246 | 21.773 | 73.203 | 74091.178 | 0.00098801 |
| 29.143 | 20.073 | 156.241 | 252196.701 | 0.00061952 |
| 29.732 | 20.399 | 104.223 | 199387.044 | 0.00052272 |
| 28.884 | 20.913 | 186.594 | 137632.382 | 0.00135574 |
| 29.384 | 21.281 | 132.377 | 105577.137 | 0.00125384 |
| 30.244 | 22.183 | 73.323 | 55114.400 | 0.00133038 |
| 29.039 | 20.832 | 145962.634 | 167.741 | 0.00114920 |
| 28.499 | 20.975 | 131647.471 | 243.240 | 0.00184766 |
| 27.476 | 21.521 | 88792.971 | 490.952 | 0.00552917 |
| 30.965 | 21.959 | 44.678 | 64797.553 | 0.00068951 |
| 30.014 | 20.873 | 85.865 | 141662.596 | 0.00060612 |

| | | | | |
|---|---|---|---|---|
| 31.131 | 23.188 | 39.861 | 26722.937 | 0.00149165 |
| viro-9(vir16); Psur-5::viro-9 (1) | | | | |
| RNA2 CT | rps-20 CT | RNA2 copy number | rps-20 copy number | Orsay virus RNA2/rps-20 copy number ratio |
| 21.714 | 22.412 | 25741.768 | 46744.938 | 0.55068569 |
| 18.559 | 20.963 | 224857.173 | 132826.476 | 1.69286411 |
| 20.292 | 20.769 | 68361.663 | 152711.023 | 0.44765375 |
| 21.464 | 22.176 | 30553.328 | 55380.588 | 0.55169743 |
| 19.933 | 21.480 | 87496.893 | 91464.686 | 0.95661940 |
| 20.663 | 21.571 | 52994.914 | 85678.561 | 0.61853179 |
| 15.135 | 20.830 | 146193.559 | 2364857.651 | 16.17620960 |
| 16.119 | 20.812 | 148015.294 | 1202220.484 | 8.12227202 |
| 17.231 | 20.304 | 213520.399 | 560184.863 | 2.62356602 |
| 17.180 | 20.121 | 580065.579 | 243562.085 | 2.38159227 |
| 16.315 | 20.856 | 1050674.960 | 143465.730 | 7.32352568 |
| 16.640 | 20.482 | 840742.273 | 187849.960 | 4.47560529 |
| viro-9(vir16); Psur-5::viro-9 (2) | | | | |
| RNA2 CT | rps-20 CT | RNA2 copy number | rps-20 copy number | Orsay virus RNA2/rps-20 copy number ratio |
| 18.718 | 20.636 | 201675.620 | 168090.061 | 1.19980693 |
| 25.954 | 23.378 | 1397.326 | 23291.472 | 0.05999304 |
| 21.109 | 22.946 | 38992.109 | 31807.519 | 1.22587709 |
| 19.008 | 21.172 | 165171.673 | 114248.863 | 1.44571831 |
| 21.055 | 21.740 | 40473.931 | 75856.235 | 0.53356103 |
| 18.460 | 21.183 | 240735.882 | 113313.393 | 2.12451393 |
| 14.906 | 21.367 | 99271.724 | 2767409.729 | 27.87711953 |
| 15.248 | 20.901 | 138834.829 | 2187788.293 | 15.75820928 |
| 14.995 | 20.615 | 170686.259 | 2602847.829 | 15.24931087 |
| 17.509 | 21.000 | 462744.736 | 129268.866 | 3.57970754 |
| 17.728 | 21.071 | 398043.430 | 122807.791 | 3.24119038 |
| 16.492 | 21.405 | 930576.988 | 96555.088 | 9.63778303 |

Exogenous Orsay virus

| Parent | | | | |
|---|---|---|---|---|
| RNA2 CT | rps-20 CT | RNA2 copy number | rps-20 copy number | Orsay virus RNA2/rps-20 copy number ratio |
| 14.161 | 21.309 | 4618260.963 | 103472.526 | 44.63272639 |
| 13.988 | 21.485 | 5199784.897 | 91123.962 | 57.06276109 |

| | | | | |
|---|---|---|---|---|
| 14.173 | 21.375 | 4578673.809 | 98702.946 | 46.38842099 |
| 18.928 | 23.186 | 174563.194 | 26760.911 | 6.52306619 |
| 17.509 | 23.056 | 462621.646 | 29382.799 | 15.74464158 |
| 16.641 | 22.494 | 840333.600 | 44066.671 | 19.06959566 |
| 15.869 | 22.251 | 1427772.349 | 52500.519 | 27.19539477 |
| 16.831 | 23.110 | 737462.943 | 28253.611 | 26.10154702 |
| 15.558 | 22.263 | 1768438.482 | 52043.881 | 33.97975780 |
| 15.956 | 23.066 | 1345360.831 | 29169.787 | 46.12172255 |
| 16.065 | 23.678 | 1247728.730 | 18772.676 | 66.46515128 |
| 15.358 | 22.787 | 2028742.507 | 35673.007 | 56.87052058 |
| viro-9(vir16) | | | | |
| RNA2 CT | rps-20 CT | RNA2 copy number | rps-20 copy number | Orsay virus RNA2/rps-20 copy number ratio |
| 27.511 | 21.457 | 479.486 | 92996.195 | 0.00515598 |
| 28.943 | 21.012 | 179.207 | 128214.994 | 0.00139770 |
| 28.159 | 20.557 | 307.104 | 177933.755 | 0.00172594 |
| 30.333 | 22.088 | 68.948 | 59017.480 | 0.00116826 |
| 30.906 | 22.668 | 46.520 | 38862.996 | 0.00119702 |
| 30.799 | 21.614 | 50.053 | 83062.517 | 0.00060260 |
| 28.888 | 20.614 | 186.119 | 170821.461 | 0.00108955 |
| 30.157 | 21.085 | 77.807 | 121588.781 | 0.00063992 |
| 29.565 | 20.935 | 116.869 | 135456.245 | 0.00086278 |
| 30.202 | 23.082 | 75.460 | 28831.929 | 0.00261725 |
| 30.983 | 24.650 | 44.135 | 9313.215 | 0.00473894 |
| 29.838 | 22.457 | 96.887 | 45254.727 | 0.00214092 |
| viro-9(vir16); Psur-5::viro-9 (1) | | | | |
| RNA2 CT | rps-20 CT | RNA2 copy number | rps-20 copy number | Orsay virus RNA2/rps-20 copy number ratio |
| 14.683 | 21.399 | 3225934.335 | 96951.963 | 33.27353306 |
| 13.697 | 21.276 | 6349717.954 | 105992.735 | 59.90710545 |
| 15.011 | 21.812 | 2575245.994 | 72020.248 | 35.75724974 |
| 16.360 | 21.656 | 1019181.461 | 80567.095 | 12.65009576 |
| 18.384 | 24.780 | 253645.224 | 8479.216 | 29.91375787 |
| 16.506 | 22.413 | 921852.076 | 46697.091 | 19.74110293 |
| 15.934 | 22.418 | 1365290.156 | 46543.029 | 29.33393441 |
| 16.570 | 22.424 | 882172.752 | 46346.897 | 19.03412752 |
| 15.199 | 21.254 | 2262121.073 | 107673.182 | 21.00914118 |
| 16.337 | 22.640 | 1035047.116 | 39639.564 | 26.11146577 |
| 15.850 | 22.523 | 1446740.437 | 43145.151 | 33.53193550 |

| | | | | |
|---|---|---|---|---|
| 16.312 | 22.164 | 1053279.210 | 55873.953 | 18.85098796 |
| viro-9(vir16); Psur-5::viro-9 (2) | | | | |
| RNA2 CT | rps-20 CT | RNA2 copy number | rps-20 copy number | Orsay virus RNA2/rps-20 copy number ratio |
| 18.890 | 23.781 | 179123.929 | 17425.299 | 10.27953273 |
| 18.139 | 21.992 | 300184.622 | 63235.636 | 4.74707995 |
| 16.449 | 21.828 | 958726.748 | 71187.348 | 13.46765641 |
| 17.533 | 23.100 | 455139.523 | 28463.336 | 15.99037861 |
| 17.244 | 21.681 | 555137.799 | 79169.830 | 7.01198680 |
| 16.680 | 22.557 | 818074.081 | 42109.092 | 19.42749258 |
| 16.762 | 22.160 | 773225.149 | 56051.812 | 13.79482882 |
| 14.620 | 21.985 | 3369059.729 | 63573.112 | 52.99504199 |
| 15.074 | 21.497 | 2466270.231 | 90379.330 | 27.28798987 |
| 14.796 | 21.919 | 2984976.396 | 66669.227 | 44.77292654 |
| 14.392 | 22.446 | 3938854.523 | 45611.776 | 86.35608777 |
| 15.141 | 21.970 | 2355204.802 | 64285.457 | 36.63666583 |
| Input | | | | |
| RNA2 CT | rps-20 CT | RNA2 copy number | rps-20 copy number | Orsay virus RNA2/rps-20 copy number ratio |
| 26.592 | 21.042 | 901.758 | 125471.145 | 0.00718697 |
| 26.295 | 21.329 | 1105.666 | 101997.893 | 0.01084008 |
| 26.476 | 21.135 | 976.556 | 117297.290 | 0.00832548 |
| 29.100 | 21.612 | 160.882 | 83184.209 | 0.00193404 |
| 28.855 | 21.511 | 190.350 | 89430.808 | 0.00212846 |
| 28.308 | 21.149 | 277.304 | 116158.364 | 0.00238729 |
| 27.299 | 21.082 | 554.552 | 121879.786 | 0.00455000 |
| 27.458 | 20.717 | 497.152 | 158533.580 | 0.00313594 |
| 27.323 | 20.734 | 545.639 | 156643.960 | 0.00348330 |
| 28.388 | 21.202 | 262.477 | 111749.962 | 0.00234879 |
| 28.598 | 21.293 | 227.098 | 104713.390 | 0.00216876 |
| 28.732 | 22.127 | 207.219 | 57384.912 | 0.00361103 |

Figure 3A. qRT-PCR quantification of Orsay virus RNA2 copy number upon infection with exogenous Orsay virus.

| rde-1 | | | | |
|---|---|---|---|---|
| RNA2 CT | rps-20 CT | RNA2 copy number | rps-20 copy number | Orsay virus RNA2/rps-20 copy number ratio |
| 12.396 | 22.509 | 43584.361 | 63765074.171 | 1463.02649207 |
| 12.435 | 22.207 | 54185.441 | 62026239.274 | 1144.70304679 |

| | | | | |
|---|---|---|---|---|
| 12.505 | 21.984 | 63603.618 | 58959891.362 | 926.98958023 |
| 18.517 | 20.239 | 773959.721 | 223705.061 | 3.45973273 |
| 17.499 | 19.833 | 1611724.375 | 299722.505 | 5.37738858 |
| 17.984 | 19.745 | 1136469.499 | 319526.308 | 3.55673217 |
| 13.031 | 23.482 | 40349077.269 | 21620.153 | 1866.27160854 |
| 13.442 | 24.057 | 30017297.980 | 14284.599 | 2101.37484971 |
| 14.272 | 25.060 | 16502250.667 | 6933.610 | 2380.03739141 |
| N2 | | | | |
| RNA2 CT | rps-20 CT | RNA2 copy number | rps-20 copy number | Orsay virus RNA2/rps-20 copy number ratio |
| 19.964 | 22.467 | 29776.476 | 272782.584 | 9.16100952 |
| 23.690 | 23.114 | 18047.788 | 18600.623 | 1.03063173 |
| 20.156 | 22.558 | 39843.662 | 237623.828 | 5.96390526 |
| 23.388 | 19.909 | 23125.925 | 283917.956 | 0.08145285 |
| 26.544 | 20.458 | 2378.390 | 191018.739 | 0.01245108 |
| 25.611 | 19.796 | 4659.948 | 307840.806 | 0.01513752 |
| 18.912 | 22.638 | 582166.562 | 39698.675 | 14.66463450 |
| 19.621 | 23.709 | 349393.699 | 18351.246 | 19.03923546 |
| 22.941 | 22.931 | 31925.223 | 32149.295 | 0.99303024 |
| viro-9(vir19) | | | | |
| RNA2 CT | rps-20 CT | RNA2 copy number | rps-20 copy number | Orsay virus RNA2/rps-20 copy number ratio |
| 33.736 | 21.929 | 66194.002 | 13.344 | 0.00020159 |
| 34.436 | 22.020 | 61982.403 | 8.054 | 0.00012995 |
| 32.328 | 20.968 | 132309.117 | 36.807 | 0.00027819 |
| 34.741 | 22.955 | 6.468 | 31598.683 | 0.00020471 |
| 33.457 | 22.660 | 16.313 | 39098.284 | 0.00041723 |
| 31.565 | 21.830 | 63.796 | 71071.971 | 0.00089763 |
| 33.176 | 22.850 | 19.979 | 34090.738 | 0.00058606 |
| 32.833 | 21.627 | 25.576 | 82272.738 | 0.00031087 |
| 31.196 | 22.766 | 83.229 | 36210.993 | 0.00229845 |
| Input | | | | |
| RNA2 CT | rps-20 CT | RNA2 copy number | rps-20 copy number | Orsay virus RNA2/rps-20 copy number ratio |
| 27.084 | 23.037 | 44925.106 | 1612.059 | 0.03588326 |
| 27.176 | 23.732 | 28179.333 | 1508.368 | 0.05352745 |
| 26.989 | 22.633 | 42075.128 | 1726.260 | 0.04102803 |
| 31.266 | 19.551 | 79.118 | 367288.210 | 0.00021541 |
| 31.195 | 18.980 | 83.278 | 554573.231 | 0.00015017 |

| | | | | |
|---|---|---|---|---|
| 29.309 | 18.658 | 324.363 | 699199.981 | 0.00046391 |
| 27.494 | 22.924 | 1199.813 | 32317.360 | 0.03712597 |
| 27.341 | 22.330 | 1339.539 | 49585.370 | 0.02701480 |
| 26.661 | 21.833 | 2185.705 | 70921.090 | 0.03081883 |

Figure 4B. qRT-PCR quantification of Orsay virus load in CBG23913-transgenic animals upon exogenous Orsay virus infection or initiation of replication from the Orsay virus transgene by heat shock.

Heat shock

| Parent | | | | |
|---|---|---|---|---|
| RNA2 CT | rps-20 CT | RNA2 copy number | rps-20 copy number | Orsay virus RNA2/rps-20 copy number ratio |
| 18.825 | 22.790 | 187392.086 | 35600.946 | 5.26368275 |
| 18.402 | 22.348 | 250585.401 | 48956.104 | 5.11857313 |
| 20.095 | 22.138 | 78262.197 | 56920.991 | 1.37492682 |
| 18.755 | 19.601 | 196575.721 | 354367.592 | 0.55472263 |
| 19.253 | 21.725 | 139569.070 | 76676.169 | 1.82024053 |
| 18.275 | 21.699 | 273389.394 | 78111.737 | 3.49997843 |
| 17.918 | 21.191 | 349385.956 | 112660.135 | 3.10123856 |
| 18.921 | 22.397 | 175429.627 | 47226.894 | 3.71461281 |
| 17.705 | 21.314 | 404327.745 | 103095.858 | 3.92186216 |
| 17.409 | 21.224 | 495574.269 | 110058.829 | 4.50281250 |
| 18.329 | 20.885 | 263387.031 | 140451.296 | 1.87529086 |
| 17.839 | 19.137 | 368724.173 | 495178.860 | 0.74462826 |
| viro-9(vir16) | | | | |
| RNA2 CT | rps-20 CT | RNA2 copy number | rps-20 copy number | Orsay virus RNA2/rps-20 copy number ratio |
| 31.493 | 22.297 | 31.078 | 50780.660 | 0.00061201 |
| 31.344 | 21.861 | 34.431 | 69541.539 | 0.00049512 |
| 31.049 | 21.842 | 42.158 | 70485.188 | 0.00059811 |
| 28.632 | 19.431 | 221.971 | 400584.070 | 0.00055412 |
| 29.647 | 22.129 | 110.511 | 57300.018 | 0.00192864 |
| 29.415 | 21.403 | 129.560 | 96695.882 | 0.00133987 |
| 31.285 | 20.074 | 35.866 | 252010.603 | 0.00014232 |
| 30.246 | 20.284 | 73.221 | 216691.947 | 0.00033790 |
| 30.425 | 21.607 | 64.727 | 83494.211 | 0.00077523 |
| 27.974 | 21.541 | 348.868 | 87566.053 | 0.00398406 |
| 27.617 | 21.635 | 445.706 | 81818.674 | 0.00544748 |
| 27.653 | 21.390 | 434.981 | 97638.745 | 0.00445500 |

| viro-9(vir16); Psur-5::CBG23913 (1) | | | | |
|---|---|---|---|---|
| RNA2 CT | rps-20 CT | RNA2 copy number | rps-20 copy number | Orsay virus RNA2/rps-20 copy number ratio |
| 19.967 | 21.252 | 85501.296 | 107811.958 | 0.79305948 |
| 18.353 | 22.423 | 259102.402 | 46375.321 | 5.58707515 |
| 18.970 | 22.189 | 169514.807 | 54895.051 | 3.08797975 |
| 16.698 | 20.767 | 807730.958 | 152957.460 | 5.28075555 |
| 17.410 | 20.623 | 495382.066 | 169638.572 | 2.92022069 |
| 18.700 | 20.105 | 204193.607 | 246418.562 | 0.82864539 |
| 19.788 | 24.083 | 96678.187 | 14015.808 | 6.89779598 |
| 19.687 | 21.140 | 103610.612 | 116881.542 | 0.88645829 |
| 18.140 | 21.106 | 299892.469 | 119768.181 | 2.50394109 |
| 23.142 | 24.243 | 9648.938 | 12492.966 | 0.77234966 |
| 20.724 | 21.519 | 50826.096 | 88939.929 | 0.57146544 |
| 20.623 | 21.408 | 54474.925 | 96338.056 | 0.56545593 |
| viro-9(vir16); Psur-5::CBG23913 (2) | | | | |
| RNA2 CT | rps-20 CT | RNA2 copy number | rps-20 copy number | Orsay virus RNA2/rps-20 copy number ratio |
| 20.601 | 23.161 | 55283.875 | 27244.034 | 2.02921029 |
| 20.162 | 21.578 | 74777.532 | 85216.360 | 0.87750207 |
| 20.532 | 23.143 | 57992.444 | 27599.493 | 2.10121409 |
| 18.200 | 20.040 | 287745.287 | 258347.804 | 1.11379033 |
| 18.040 | 18.664 | 321319.127 | 696216.237 | 0.46152202 |
| 18.463 | 22.294 | 240168.040 | 50881.209 | 4.72017166 |
| 18.880 | 21.005 | 180374.068 | 128842.917 | 1.39995331 |
| 17.409 | 22.405 | 495778.892 | 46953.790 | 10.55886846 |
| 17.818 | 22.660 | 374153.611 | 39083.345 | 9.57322378 |
| 22.890 | 21.880 | 11472.011 | 68559.236 | 0.16732991 |
| 18.567 | 21.563 | 223681.535 | 86202.379 | 2.59484177 |
| 21.309 | 23.400 | 33998.552 | 22934.836 | 1.48239785 |

Exogenous Orsay virus

| Parent | | | | |
|---|---|---|---|---|
| RNA2 CT | rps-20 CT | RNA2 copy number | rps-20 copy number | Orsay virus RNA2/rps-20 copy number ratio |
| 16.769 | 22.269 | 769330.632 | 51808.329 | 14.84955504 |
| 16.864 | 22.155 | 720901.029 | 56226.836 | 12.82129814 |
| 16.131 | 23.178 | 1192446.409 | 26912.421 | 44.30840385 |

| | | | | |
|---|---|---|---|---|
| 13.968 | 20.764 | 5271292.179 | 153247.054 | 34.39734766 |
| 13.572 | 21.511 | 6920183.505 | 89457.858 | 77.35691017 |
| 14.945 | 22.380 | 2693897.531 | 47839.326 | 56.31136096 |
| 15.315 | 22.757 | 2088598.718 | 36458.731 | 57.28665473 |
| 14.286 | 21.397 | 4237493.385 | 97113.759 | 43.63432575 |
| 14.908 | 21.712 | 2763982.757 | 77389.773 | 35.71509060 |
| 15.108 | 19.790 | 2409046.271 | 309186.456 | 7.79156468 |
| 14.108 | 21.559 | 4786785.272 | 86445.641 | 55.37335616 |
| 14.066 | 20.890 | 4928909.592 | 139986.382 | 35.20992189 |
| viro-9(vir16) | | | | |
| RNA2 CT | rps-20 CT | RNA2 copy number | rps-20 copy number | Orsay virus RNA2/rps-20 copy number ratio |
| 30.609 | 21.046 | 57.038 | 125070.947 | 0.00045604 |
| 30.854 | 21.344 | 48.216 | 100871.818 | 0.00047799 |
| 30.551 | 20.634 | 59.388 | 168379.601 | 0.00035270 |
| 28.733 | 20.570 | 207.043 | 176299.344 | 0.00117439 |
| 30.108 | 22.082 | 80.475 | 59265.846 | 0.00135787 |
| 29.677 | 21.562 | 108.241 | 86262.360 | 0.00125478 |
| 28.248 | 21.962 | 288.939 | 64633.510 | 0.00447041 |
| 27.489 | 21.184 | 486.586 | 113277.261 | 0.00429553 |
| 28.001 | 22.980 | 342.441 | 31027.119 | 0.01103682 |
| 28.998 | 21.796 | 172.614 | 72864.696 | 0.00236897 |
| 28.277 | 20.218 | 283.322 | 227165.246 | 0.00124721 |
| 28.880 | 19.982 | 187.180 | 269364.511 | 0.00069489 |
| viro-9(vir16); Psur-5::CBG23913 (1) | | | | |
| RNA2 CT | rps-20 CT | RNA2 copy number | rps-20 copy number | Orsay virus RNA2/rps-20 copy number ratio |
| 15.467 | 21.853 | 1882432.794 | 69939.871 | 26.91501673 |
| 16.252 | 20.975 | 1097281.747 | 131674.076 | 8.33331648 |
| 14.881 | 21.229 | 2814799.126 | 109662.405 | 25.66785873 |
| 13.885 | 20.226 | 5579554.704 | 225866.804 | 24.70285406 |
| 14.664 | 21.249 | 3267769.640 | 108021.720 | 30.25104248 |
| 15.879 | 22.316 | 1418424.056 | 50071.689 | 28.32786493 |
| 15.166 | 21.131 | 2314289.484 | 117621.995 | 19.67565234 |
| 15.592 | 21.600 | 1727702.638 | 83924.803 | 20.58631744 |
| 14.202 | 21.029 | 4488292.819 | 126597.968 | 35.45311886 |
| 15.853 | 21.050 | 1443249.405 | 124723.278 | 11.57161217 |
| 15.558 | 23.062 | 1767685.429 | 29261.997 | 60.40891205 |
| 15.829 | 23.323 | 1467764.650 | 24245.696 | 60.53712206 |

| viro-9(vir16); Psur-5::CBG23913 (2) | | | | |
|---|---|---|---|---|
| RNA2 CT | rps-20 CT | RNA2 copy number | rps-20 copy number | Orsay virus RNA2/rps-20 copy number ratio |
| 16.965 | 21.744 | 672601.428 | 75609.714 | 8.89570131 |
| 15.464 | 21.246 | 1885737.733 | 108287.846 | 17.41412177 |
| 15.989 | 22.667 | 1314986.491 | 38879.988 | 33.82167944 |
| 15.096 | 21.806 | 2428832.184 | 72325.223 | 33.58209029 |
| 13.592 | 21.447 | 6825941.546 | 93709.816 | 72.84126494 |
| 15.248 | 21.374 | 2187804.062 | 98715.294 | 22.16276710 |
| 14.674 | 21.368 | 3245940.102 | 99172.429 | 32.73026711 |
| 15.365 | 21.468 | 2018848.382 | 92308.816 | 21.87059112 |
| 15.138 | 21.939 | 2359189.819 | 65733.008 | 35.89048946 |
| 16.610 | 21.060 | 857934.276 | 123782.331 | 6.93099145 |
| 16.519 | 20.493 | 913346.608 | 186312.611 | 4.90222644 |
| 15.025 | 21.989 | 2549179.321 | 63394.824 | 40.21115833 |
| Input | | | | |
| RNA2 CT | rps-20 CT | RNA2 copy number | rps-20 copy number | Orsay virus RNA2/rps-20 copy number ratio |
| 28.880 | 22.977 | 187.193 | 31100.651 | 0.00601893 |
| 29.054 | 22.134 | 166.043 | 57101.084 | 0.00290788 |
| 27.566 | 21.037 | 461.691 | 125873.835 | 0.00366789 |
| 27.353 | 21.469 | 534.469 | 92208.880 | 0.00579629 |
| 26.873 | 21.661 | 743.302 | 80279.547 | 0.00925892 |
| 25.440 | 20.575 | 1989.955 | 175696.688 | 0.01132608 |
| 26.589 | 21.741 | 903.462 | 75790.258 | 0.01192056 |
| 25.993 | 21.604 | 1360.223 | 83635.733 | 0.01626366 |
| 25.934 | 20.979 | 1416.345 | 131301.915 | 0.01078694 |
| 27.720 | 21.896 | 415.253 | 67807.456 | 0.00612400 |
| 28.773 | 22.800 | 201.378 | 35320.854 | 0.00570138 |
| 26.082 | 19.849 | 1279.776 | 296375.214 | 0.00431809 |

Figure S1. qRT-PCR quantification of Orsay virus RNA2 in the viro-9(sy1174) obtained from the CGC.

Exogenous Orsay virus

| N2 | | | | |
|---|---|---|---|---|
| RNA2 CT | rps-20 CT | RNA2 copy number | rps-20 copy number | Orsay virus RNA2/rps-20 copy number ratio |
| 19.964 | 23.037 | 29776.476 | 272782.584 | 9.16100952 |

| | | | | |
|---|---|---|---|---|
| 23.690 | 23.732 | 18047.788 | 18600.623 | 1.03063173 |
| 20.156 | 22.633 | 39843.662 | 237623.828 | 5.96390526 |
| 23.388 | 19.909 | 23125.925 | 283917.956 | 0.08145285 |
| 26.544 | 20.458 | 2378.390 | 191018.739 | 0.01245108 |
| 25.611 | 19.796 | 4659.948 | 307840.806 | 0.01513752 |
| 18.912 | 22.638 | 582166.562 | 39698.675 | 14.66463450 |
| 19.621 | 23.709 | 349393.699 | 18351.246 | 19.03923546 |
| 22.941 | 22.931 | 31925.223 | 32149.295 | 0.99303024 |
| viro-9(sy1174) | | | | |
| RNA2 CT | rps-20 CT | RNA2 copy number | rps-20 copy number | Orsay virus RNA2/rps-20 copy number ratio |
| 34.692 | 22.094 | 58785.102 | 6.699 | 0.00011395 |
| 32.198 | 20.568 | 176565.158 | 40.411 | 0.00022887 |
| 31.857 | 20.162 | 236495.013 | 51.700 | 0.00021861 |
| 31.466 | 19.749 | 68.491 | 318556.638 | 0.00021500 |
| 30.296 | 18.210 | 159.252 | 965630.876 | 0.00016492 |
| 37.810 | 19.038 | 0.708 | 531752.904 | 0.00000133 |
| 30.331 | 22.765 | 155.265 | 36226.377 | 0.00428596 |
| 33.786 | 22.905 | 12.868 | 32754.632 | 0.00039287 |
| 32.937 | 22.598 | 23.725 | 40881.488 | 0.00058034 |
| Input | | | | |
| RNA2 CT | rps-20 CT | RNA2 copy number | rps-20 copy number | Orsay virus RNA2/rps-20 copy number ratio |
| 27.084 | 22.467 | 44925.106 | 1612.059 | 0.03588326 |
| 27.176 | 23.114 | 28179.333 | 1508.368 | 0.05352745 |
| 26.989 | 22.558 | 42075.128 | 1726.260 | 0.04102803 |
| 31.266 | 19.551 | 79.118 | 367288.210 | 0.00021541 |
| 31.195 | 18.980 | 83.278 | 554573.231 | 0.00015017 |
| 29.309 | 18.658 | 324.363 | 699199.981 | 0.00046391 |
| 27.494 | 22.924 | 1199.813 | 32317.360 | 0.03712597 |
| 27.341 | 22.330 | 1339.539 | 49585.370 | 0.02701480 |
| 26.661 | 21.833 | 2185.705 | 70921.090 | 0.03081883 |
